# Exploring ideal scent detection dog characteristics for successful professional deployment as derived from face-to-face structured interviews with professional scent detection dog handlers

**DOI:** 10.1371/journal.pone.0339379

**Published:** 2026-02-04

**Authors:** Pascalle E. M. Roulaux, Claudia M. Vinke, Ineke R. van Herwijnen, Saskia S. Arndt

**Affiliations:** Division of Animals in Science and Society, Department of Population Health Sciences, Faculty of Veterinary Medicine, Utrecht University, Utrecht, The Netherlands; Texas Christian University, UNITED STATES OF AMERICA

## Abstract

Scent detection dogs (SDDs) require certain behavioral characteristics for successful professional deployment. We aimed to provide insight into these characteristics by tapping into professional SDD handlers’ knowledge and experience. We conducted a first round of informal interviews with Dutch professional SDD handlers, which led to the derivation of a list of 24 characteristics. We subsequently used this list in structured interviews and had participants (N = 110) indicate how their ‘ideal SDD’ would score on a scale from 0 to 100 (ideal scores) and how reluctant they would be to see an actual dog deviate from their ideal image for each characteristic (allowed deviation scores). We compared the allowed deviation scores to the variation in the ideal scores. Additionally, we compared ideal dog characteristics for professional deployment versus recreational deployment by civilians to assess how these characteristics are relevant to professional deployment of SDDs. Professional participants were the most reluctant to make concessions for these SDD characteristics: *neurotic, insecure, imperturbable (environment), scent-oriented, independent, confident, persistent,* and *resilient*. Seven of these eight characteristics were also among those with the least variation in ideal scores between professionals. Four ‘ideal SDD’ characteristics differed significantly between civilians and professionals: civilians desired lower scores for *unreserved*, *impulsive*, *excited* and *possessive*. Three allowed deviation scores differed significantly: professionals were more reluctant to make concessions for *unreserved*, while civilians were more reluctant to make concessions for *precise* and *possessive*. Focusing future on the characteristics that professional handlers are reluctant to see deviate from their ideal image could enhance SDD selection and training for professional deployment and provide a basis for the development of behavioral tests or specific observations for professional SDD selection in the future. We pinpoint the characteristics of *neurotic*, *insecure*, *imperturbable (environment), scent-oriented*, *independent*, *confident*, *persistent* and *resilient* as possible focus points in such research.

## Introduction

Scent detection dogs (SDDs) have been trained and deployed by humans for over two centuries to search for a variety of odors, such as human scents, explosives, narcotics or currency [1]. Precise numbers of worldwide professional SDDs are unavailable and may even be classified, depending on the field in which the SDDs are active. However, for frame of reference, even a relatively small country as the Netherlands has hundreds of active governmental SDDs (owned by the Dutch government and deployed professionally). Alternatives to SDDs, like electronic scent detection tools, the so-called ‘electronic noses’, are in relatively early stages of development [2] and presently considered unable to equal the sensitivity and specificity of SDDs [3]. For example, 92.1% sensitivity and 97.4% specificity was reached for the detection of black tea by dogs, with only two days of training [4]. Hidden narcotics were found by fully trained police dogs after 64 seconds’ searching time, with 87.7% indications being correct and 5.3% being false (in 7.0% of the trials, the dogs failed to find the drug sample within 10 minutes) [5]. These results were similar to those of earlier studies providing data on sensitivity and specificity of scent detection dogs (sensitivity 72–100%, specificity 91–99%) [4]. In contrast, electronic nose sensitivity ranged from 48.3% to 95.8% and specificity from 10.0% to 100.0% in a systematic review of 52 studies on the cancer diagnosis performance of electronic noses using exhaled breath [6]. However, the sensitivity and specificity of electronic noses may vary, perhaps depending on the odor source, as 95% sensitivity and 90% specificity was reported in the detecting cannabis use on the human skin surface [7].

Due to their consistently high sensitivity and specificity during working tasks, SDDs are still deployed in roles that are critically important for society, such as finding missing persons, preventing explosions and disrupting drug trafficking. The demand for successfully performing professional SDDs is therefore likely to remain high; at least the coming decennia. A notorious complication in meeting this demand is the generally low percentage of dogs suitable for professional deployment. In Japan for example, only 30% of the dogs that enter the training program become professional drug detection dogs [8] and only 1.3% of 1310 German Shepherds and 10.9% of 797 Labrador Retrievers were deemed suitable as SDDs in Sweden [9]. In the latter study, the dogs were either bred at the Swedish Dog Training Centre or purchased from private breeders and about 50% of the dogs that were initially selected for training, were disqualified during the training period. Selecting unsuitable dogs that later fail the training programs can have economical consequences, as training and housing entail costs. Also, there may be animal welfare consequences when unsuitable dogs are unable to adjust to the demands of their work [10]. Particularly, as these dogs were selected for specific purposes, their chances of being rehomed may be smaller [11]. Also, when relinquished to a shelter environment, these dogs may be challenged to adapt to the particularities of this environment, as seen in high percentages of kenneled working dogs showing repetitive behavior [12]. Enhancing the selection of suitable dogs, therefore, may have both animal welfare and financial costs benefits. Having a better insight into behavioral characteristics that are deemed essential for successful professional deployment by experienced professional SDD handlers and trainers is at the basis of enhanced selection, whether dogs are selected as puppies or – like in The Netherlands – as adolescent dogs.

Existing research provides some insight into these essential characteristics. For instance [13] found important traits to include high play motivation, high level of cooperativeness with the handler, boldness, obedience yet independence when off-leash, and high athleticism. In the present study, we aim to provide further insight by interviewing a study population that was not previously studied: professional SDD handlers and trainers and a control group of civilian dog handlers on what constitutes ‘their ideal SDD’, without guiding them in any direction beforehand. We then determined: a) which surveyed ‘ideal dog’ characteristics are scored with the least variation, as these might be of particular interest for dog selection, b) which characteristics participants would be most reluctant to see deviate from their ideal image when selecting an actual dog, and c) ideal dog characteristics for professional deployment of the dogs versus recreational deployment by civilians, to elucidate the typically needed professional characteristics. We expect the differences between the professionals and civilians to highlight the characteristics that are needed for professional deployment, versus those that are also present in pet dogs that practice detection with their owners for recreational purposes. We hypothesize professional participants to be more reluctant to see characteristics that were scored with less variation deviate from their ideal image, than characteristics that were scored with more variation. We furthermore hypothesize civilian handlers to less frequently score characteristics at the extremes, i.e., be ‘less demanding’; thus resulting in higher means for generally unwanted characteristics and lower means for generally wanted characteristics. Insight into which characteristics are essential for ‘the ideal SDD’ for professional deployment could provide a basis for the development of behavioral tests and/or specific observations for professional SDD selection in the future.

## Methods

### Informal interviews

To identify SDD characteristics that are potentially relevant to handlers and trainers, we conducted informal interviews (as described by Bernard [14]) with experienced Dutch professional SDD handlers and trainers. This led to a list of 24 SDD characteristics, with a short elucidation, in order to standardize the interpretation (see Table 1). The list contained both strongly wanted and unwanted characteristics, as mentioned by the interviewees.

**Table 1:** SDD characteristics and elucidation (translated from Dutch).

Characteristic	Explanation
Dependent	Strongly attached to and seeks support from the handler.
Alert	Watchful, attentive.
Possessive	Does not like to give up possessions.
Balanced	Evenly; behaves consistently in similar situations.
Flexible	Easily switches between work and rest.
Handler-oriented	Likes to work together with the handler.
Scent-oriented	Has a strong preference to search by scent rather than sight.
Sensitive	Easily affected by stimuli from the environment or administered by the handler.
Impulsive	Uninhibited; acts spontaneously and quickly.
Calm	Quiet; not easily excited.
Eager to learn	Learns new things quickly and easily.
Precise	Accurate, with attention to detail.
Neurotic	Strongly inclined to show stress and/or negative emotions such as fear or frustration.
Unreserved	Free from fear and anxiety.
Imperturbable (environment)	Is not easily distracted by environmental factors such as traffic or food that was not offered.
Imperturbable (social)	Is not easily distracted by unfamiliar people and animals.
Insecure	Hesitates; is full of doubt; acts with delay.
Excited	Restless; finds it hard to be patient.
Resilient	Easily recovers from stress and adversity.
Docile	Immediately follows instructions from the handler.
Persistent	Perseveres despite setbacks and obstacles.
Careful	Thoughtful; thinks before acting.
Independent	Can easily complete tasks independent from the handler.
Confident	Full of self confidence; purposeful.

### Participants and participant recruitment method

The data from 110 face-to-face structured interviews were included, of which 65 were conducted with handlers (N = 53) and trainers (N = 12) from the Dutch police, 22 with handlers (N = 19) and trainers (N = 3) from a second professional organization and 23 with civilian handlers. The dogs of the handlers in the control group perform the same tasks of scent detection, but in a recreational setting instead of a professional setting. Thus, the study included all of the SDD handlers and trainers of the Dutch police organization, with the exception of three (4%) who were unavailable. Both professional organizations employ handlers and trainers. Trainers select and train young dogs, after which they match them to a handler and train the team up to their certification. They are also responsible for maintenance and proficiency training of the teams. Handlers work with the dogs operationally and are responsible for the day to day training and husbandry of their dogs. In some cases dogs later switch to another handler, but the premise is that after general training, each dog has one permanent handler. The majority of the professional handlers and trainers work with Belgian Malinois or similar Shepherd breeds (>95%). Dogs are of varying ages and both sexes were included.

The 23 civilian handlers, who practice detection as a sport with their pet dog served as a control group. These handlers were recruited through Argos Scentwork Academy; one of the largest non-professional-oriented scent-work organizations in the Netherlands. Their dogs are trained on, for instance, anise, clove or cinnamon. These dogs are of a large variety of breeds and ages, and include both sexes. Handlers were all advanced, i.e., they had attended at least twenty training sessions with their dog over the course of multiple months, during which they also trained at home. Most handlers had detection experience with only one dog. The dogs were all able to search and indicate independently from the handler and they could ignore mild to more strongly present distractions while doing so.

### Professional handlers’ and trainers’ scentwork disciplines

Participants from the second professional organization worked in the detection of tobacco (in cargo), currency (on person) or narcotics (in cargo or on person). Most handlers had one dog, but one handler had two dogs in two different disciplines. Trainers all worked with multiple dogs in multiple disciplines. The police had a larger variety of disciplines. Here, most handlers had two dogs, that trained in two different disciplines. Trainers worked mainly in one discipline; explosives and human scent detection being the largest ones. Handlers worked mainly in the detection of explosives, human scent, narcotics or currency. Smaller numbers of handlers and trainers worked for instance in the detection of arson, blood and human remains, or digital storage devices. We tested for possible effects of the discipline; see Results section 3.1.

### The face-to-face structured interview procedure

Professional SDD handlers and trainers and – as a control group – civilians working with their pet dogs for detection sport, filled in a survey on SDD characteristics during a face-to-face structured interview. The interviews were conducted in Dutch, which was the native language of all the participants and the first author. The set-up and procedures were standardized and first pre-tested with native speakers, both from within and outside the target population, to assess practicality and understandability.

At the start of the interview, the participants were first informed about the purpose of the research and given a brief overview of the contents of the survey by the first author. Interviews lasted 44 minutes on average (SD = 16.8). In addition to questions on dog characteristics, participants answered general questions about themselves, including their gender identity and year of birth. For the professional handlers and trainers also the number of years of professional experience were included, background in dog training, the number of dogs they trained in their profession and the discipline in which they work (See Methods section 2.3).

Subsequently, the participants were asked to imagine to be able to create *their ideal scent detection dog, as if it were a robot created in a laboratory*. They were asked how this imaginary ideal dog would score on the pre-determined list of 24 characteristics. The participant was given a standardized answer form. The interviewer read the description for each characteristic, one by one, after which the participant would indicate a score between 0 and 100, by drawing a vertical dash on the line following the characteristic (0 = preferably not present and 100 = preferably present very strongly; N.B. maximum scores are not always pursued, especially for unwanted characteristics, but also for wanted characteristics that may become a nuisance when they are present too strongly). The characteristics were alphabetized in Dutch and introduced in the same order for each participant.

Subsequently, participants were asked to indicate to which extent deviation from the *ideal dog* mattered for an *actual dog* that they selected for professional deployment. In other words, the participants were asked, for each characteristic, to which extent a deviation from the ideal dog was acceptable to them. To answer, participants divided cards containing both the characteristic and the explanation between three plastic containers. The containers were labelled ‘little deviation’, ‘average deviation’ and ‘large deviation’, referring to the extent to which the participants would allow an *actual dog* to deviate from their *ideal dog*, which we from hereon refer to as the ‘allowed deviation score’ for each characteristic. We limited each container to eight cards, thus forcing the participant to think critically about card choice and placement, as in pretesting people proved inclined to simply place all cards in the container with ‘little deviation’ without further thinking it through. As this prevented us from finding possible differences between characteristics in how these may differ in accepting deviation from an ideal dog, we asked the participants to split the cards equally over each of the three containers.

As described in the Methods section 2.3, the professional handlers and trainers worked in different disciplines and could be active in more than one discipline at the same time. In total, 35 of the professional participants were active in two or more disciplines. These participants were given the option to fill in the survey twice, if they opinioned the imaginary ideal SDD to differ depending on the discipline. Six of the participants opted for this second survey, leading to a total number of 116 entries for each characteristic. All six of these participants had SDDs in the human scent detection discipline combined with another discipline. These participants also rearranged the cards for the allowed deviation scores depending on the discipline. Another eight participants opted not for a second survey, but did rearrange the cards for the allowed deviation scores. One of these participants was active in three disciplines, resulting in nine extra entries for each characteristic for the allowed deviation scores, thus leading to a total of 125 entries.

### Data processing and analysis

The scores from the answer form were converted by measuring the score represented by the vertical dash with a ruler (the score of 0–100 being represented by measures of 0–100 mm) and then entered into an IBM SPSS Statistics 29 datafile. The 0–100 scale was regarded as continuous. The scores from the division of the cards over the three containers were converted by entering ‘little deviation’ as ‘1’, ‘average deviation’ as ‘2’ and ‘large deviation’ as ‘3’. These will further be referred to as ‘allowed deviation scores’. Statistical analyses were performed in IBM SPSS Statistics 29.

We calculated the mean values and the standard deviations for the ideal scores so as to provide an insight into the magnitude of variation per characteristic, for professionals and civilians separately. We compared the scores given by professional versus civilian handlers using independent samples t-tests. We compared the categorical allowed deviation scores using Chi-square tests. Applying the Bonferroni correction, we adjusted our alpha from 0.05 to 0.0021 (0.05/24) for all t-tests, thus we regarded p-values <0.0021 as significant.

We tested for possible effects of gender, year of birth and discipline using four separate Multivariate Analysis of Variance (MANOVA): 1) gender and year of birth as fixed factors and ideal scores for all 24 characteristics as dependent variables, 2) the discipline as fixed factor and ideal scores for all 24 characteristics as dependent variables, 3) gender and year of birth as fixed factors and 1–3 allowed deviation scores for all 24 characteristics as dependent variables, and 4) the discipline as fixed factor and 1–3 allowed deviation scores for all 24 characteristics as dependent variables. To test for effects of the discipline in which handlers were active, we included disciplines for which we had data from at least ten participants. These were explosives (N = 17), narcotics (N = 14), human scent (N = 19) and currency (N = 13) in the police organization and narcotics (N = 11) in the second professional organization.

### Ethical statement

The survey research reported in this article involves healthy human participants and does not utilize any invasive subjects, techniques, substance administration or psychological manipulations. Besides age and gender, the survey did not contain personal or sensitive information. All participants were informed of the purpose of the study and that participation was voluntary. Participants could withdraw at any moment in the process and any data that had been collected up to that point would be deleted. The research was conducted in accordance with the principles expressed in the Declaration of Helsinki and the General Data Protection Regulation. Even though data was gathered in person, it was anonymized when entered into SPSS and thus processed anonymously. All participants gave verbal consent for participation after being informed on the interview and its aim. Official signed informed consent was obtained institutionally from the Dutch police and the second professional organization and individually from all civilian handlers.

## Results

### Descriptive participant characteristics

Due to earlier described differences in the tasks of handlers and trainers, the latter had – on average – working experience with a larger number of dogs (trainers versus handlers mean number of dogs ±SD: 27.60 ± 27.18 versus 5.72 ± 5.30; independent samples t-test, P < 0.001), without there being a significant difference in their years of experience (trainers versus handlers mean ±SD: 14.80 ± 9.65 versus 11.40 ± 9.14; independent samples t-test, P = 0.198). Noteworthy was also the ratio of gender identity (with all participants identifying as either man or woman). Participants from the professional organizations were 26% (N = 23) women and 74% men (N = 64), whereas civilian handlers were 96% women (N = 22) and 4% men (N = 1). We tested for effects of gender and/or year of birth and/or number of dogs handled on the ideal scores for all characteristics using MANOVA and found no effects (See S1 File Supporting information for details). Surprisingly, we also found no effects of the discipline in which participants worked on the ideal scores for any of 24 characteristics (See S1 File Supporting information for details). We also found no effects of gender, year of birth or discipline for any of the 1–3 allowed deviation scores for any of 24 characteristics (See S1 File Supporting information for details).

### Ideal scent detection dog characteristics

**Fig 1:**
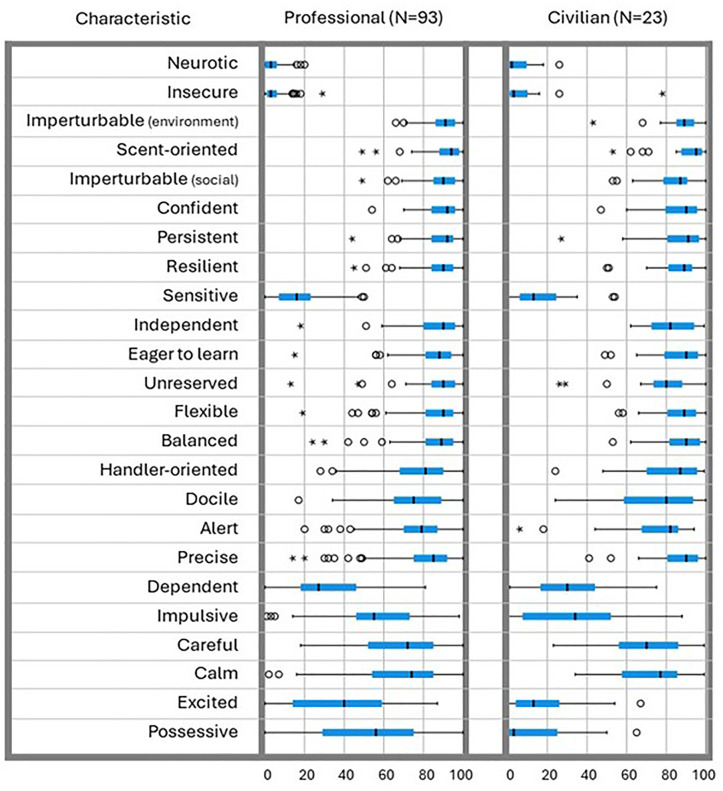
Boxplots for the ‘ideal dog’ score for each characteristic (24 in total), for professional handlers and trainers (N = 93) and civilian handlers (N = 23) separately.

To indicate which surveyed ideal scent detection dog (SDD) characteristics were scored with the least variation, we calculated mean scores ±SD for each characteristic, for professional handlers and trainers and civilian handlers separately. We sorted the characteristics by the professionals’ ascending standard deviation and we present boxplots (medians and 95% interquartile ranges) in Fig 1 and all scores in Table 2. The eight characteristics that were scored with the least variation were *neurotic, insecure, imperturbable (environment), scent-oriented, imperturbable (social), confident, persistent and resilient.* We then compared the means of all 24 characteristics between professionals and civilians using independent samples t-tests. Professionals indicated higher ideal scores than civilians for the characteristics *unreserved* (88 ± 12.9 versus 77 ± 19.0; P = 0.001), *impulsive* (56 ± 21.2 versus 34 ± 25.6; P < 0.001), *excited* (37 ± 26 versus 19 ± 18.0; P < 0.001) and *possessive* (52 ± 27 versus 14 ± 18.2; P < 0.001); thus scoring more towards the extremes than civilians only on *unreserved*. Details are presented in Table 2.

**Table 2:** Mean scores ±SD for the ideal scent detection dog (SDD) for each of the 24 characteristics, as scored by professional and civilian SDD handlers and trainers, sorted by the professionals’ SD. We then compared professional and civilian scores by independent samples t-tests (df = 114 for all), regarding P-values <0.0021 as significant, after Bonferroni correction.

Characteristic	Ideal score Mean ±SD		Independent samples t-tests
	Professional (N = 93)	Civilian (N = 23)	P-value (t-value)
Neurotic	4 ± 4.5	5 ± 7.2	0.256 (−1.142)
Insecure	4 ± 5.1	9 ± 16.3	0.209 (−1.294)
Imperturbable (environment)	90 ± 7.2	87 ± 12.2	0.091(1.703)
Scent-oriented	92 ± 8.9	89 ± 12.9	0.215 (1.247)
Imperturbable (social)	89 ± 9.1	83 ± 13.2	0.068 (1.899)
Confident	89 ± 9.4	85 ± 13.7	0.195 (1.330)
Persistent	89 ± 9.6	86 ± 16.6	0.326 (1.001)
Resilient	88 ± 10.5	85 ± 13.7	0.335 (0.967)
Sensitive	17 ± 12.0	16 ± 15.0	0.905 (0.119)
Independent	87 ± 12.5	83 ± 11.8	0.155 (1.433)
Eager to learn	86 ± 12.6	85 ± 14.8	0.712 (0.370)
Unreserved	88 ± 12.9	77 ± 19.0	**0.001 **(3.394)
Flexible	86 ± 14.1	86 ± 12.8	0.867 (0.168)
Balanced	85 ± 14.4	87 ± 12.9	0.468 (−0.729)
Handler-oriented	77 ± 17.1	80 ± 19.0	0.461 (−0.739)
Docile	75 ± 17.3	74 ± 22.4	0.938 (0.077)
Alert	76 ± 17.5	73 ± 23.0	0.403 (0.839)
Precise	80 ± 18.0	85 ± 15.3	0.196 (−1.302)
Dependent	31 ± 19.6	33 ± 22.3	0.703 (−0.383)
Impulsive	56 ± 21.2	34 ± 25.6	**<0.001 **(4.252)
Careful	68 ± 21.9	70 ± 19.8	0.651 (−0.453)
Calm	69 ± 22.7	71 ± 20.4	0.588 (−0.543)
Excited	37 ± 25.9	19 ± 18.0	**<0.001** (3.925)
Possessive	52 ± 26.5	14 ± 18.2	**<0.001 **(7.955)

### Allowed deviation from the ideal scent detection dog

Participants provided allowed deviation scores for each of the 24 characteristics, indicating if they allowed for little, average or large deviation from their ideal image when selecting an actual dog. Over 45% of the professional participants allowed little variation in the eight characteristics of *scent-oriented* (73.1%), *neurotic* (63.4%), *persistent* (62.4%), *insecure* (55.9%), *resilient* (47.3%), *confident* (45.2%), *independent* (45.2%), and *imperturbable (environment)* (45.2%). Except for *independent*, these characteristics overlap with the eight that were scored with the least variation. We then compared the counts for little, average and large deviation for all 24 characteristics between professionals and civilians using chi-square tests. Professionals were more reluctant to see an actual dog’s score for *unreserved* deviate from their ideal score, as 41.9% (N = 39) allowed little deviation, versus only 13.0% (N = 3) of the civilians (χ2 = 15.763, P < 0.001, df = 2, N = 116). Professionals were less reluctant than civilians to see an actual dog deviate from their ideal image for *precise*, as only 23.7% (N = 22) indicated allowing little variation, versus 65.2% (N = 15) of the civilians (χ2 = 15.165, P < 0.001, df = 2, N = 116). They were also less reluctant to see deviation for *possessive*, as only 11.8% (N = 11) indicated allowing little variation, versus 43.5% (N = 10) of the civilians (χ2 = 13.528, P = 0.001, df = 2, N = 116). Details on all characteristics are presented in Table 3.

**Table 3:** Allowed deviation score comparison between professional and civilian SDD handlers and trainers, for 24 SDD characteristics. Participants indicated for each characteristic if they allowed for little, average or large deviation from their ideal image when selecting an actual dog. Characteristics are sorted by descending percentage of professionals allowing little deviation, and – in the case of identical percentages for little deviation – further sorted by descending percentage of allowing average deviation. Each row represents a Chi-square test and we provide test statistics, P-values, degrees of freedom, counts, and, below the counts, the expected counts and adjusted residuals. We identify significant deviations from expected counts (i.e., adjusted residual > │2│ in bold) and we regarded P-values <0.0021 as significant, after Bonferroni correction.

		Professionals (N = 93)	Civilians (N = 23)
**Scent-oriented** χ2 = 0.597, P = 0.742, df = 2, N = 116	Little deviation	68 (73.1%) 66.5 (0.8)	15 (65.2%) 16.5 (−0.8)
	Average deviation	18 (19.4%) 19.2 (−0.7)	6 (26.0%) 4.8 (0.7)
	Large deviation	7 (7.5%) 7.2 (−0.2)	2 (8.7%) 1.8 (0.2)
**Neurotic** χ2 = 0.625, P = 0.732, df = 2, N = 116	Little deviation	59 (63.4%) 57.7 (0.6)	13 (56.6%) 14.3 (−0.6)
	Average deviation	13 (14.0%) 12.8 (0.1)	3 (13.0%) 3.2 (−0.1)
	Large deviation	21 (22.6%) 22.4 (−0.8)	7 (30.4%) 5.6 (0.8)
**Persistent** χ2 = 1.733, P = 0.420, df = 2, N = 116	Little deviation	58 (62.4%) 55.3 (1.3)	11 (47.8%) 13.7 (−1.3)
	Average deviation	25 (26.9%) 26.5 (−0.8)	8 (34.8%) 6.5 (0.8)
	Large deviation	10 (10.8%) 11.2 (−0.9)	4 (17.4%) 2.8 (0.9)
**Insecure** χ2 = 2.089, P = 0.352, df = 2, N = 116	Little deviation	52 (55.9%) 48.9 (1.4)	9 (39.1%) 12.1 (−1.4)
	Average deviation	23 (24.7%) 24.9 (−1.0)	8 (34.8%) 6.1 (1.0)
	Large deviation	18 (19.4%) 19.2 (−0.7)	6 (26.1%) 4.8 (0.7)
**Resilient** χ2 = 0.639, P = 0.727, df = 2, N = 116	Little deviation	44 (47.3%) 45.7 (−0.8)	13 (56.6%) 11.3 (0.8)
	Average deviation	40 (43.0%) 38.5 (0.7)	8 (34.8%) 9.5 (−0.7)
	Large deviation	9 (9.7%) 8.8 (0.1)	2 (8.7%) 2.2 (−0.1)
**Confident** χ2 = 6.776, P = 0.034, df = 2, N = 116	Little deviation	42 (45.2%) 38.5 (1.7)	6 (26.0%) 9.5 (−1.7)
	Average deviation	39 (41.9%) 38.5 (0.2)	9 (39.1%) 9.5 (−0.2)
	Large deviation	12 (12.9%) 16.0 (−2.5)	8 (34.8%) 4.0 (2.5)
**Independent** χ2 = 4.209, P = 0.122, df = 2, N = 116	Little deviation	42 (45.2%) 37.7 (2.0)	5 (21.7%) 9.3 (−2.0)
	Average deviation	29 (31.2%) 31.3 (−1.1)	10 (43.5%) 7.7 (1.1)
	Large deviation	22 (23.7%) 24.1 (−1.1)	8 (34.8%) 5.9 (1.1)
**Imperturbable (environment)** χ2 = 1.900, P = 0.387, df = 2, N = 116	Little deviation	42 (45.2%) 40.9 (0.5)	9 (39.1%) 10.1 (−0.5)
	Average deviation	27 (29.0%) 29.7 (−1.3)	10 (43.5%) 7.3 (1.3)
	Large deviation	24 (25.8%) 22.4 (0.8)	4 (17.4%) 5.6 (−0.8)
**Unreserved** **χ2 = 15.763, P < 0.001, df = 2, N = 116**	Little deviation	**39 (41.9%)** **33.7 (2.6)**	**3 (13.0%)** **8.3 (−2.6)**
	Average deviation	44 (47.3%) 43.3 (0.3)	10 (43.5%) 10.7 (−0.3)
	Large deviation	**10 (10.8%)** **16.0 (−3.7)**	**10 (43.5%)** **4.0 (3.7)**
**Sensitive** χ2 = 0.018, P = 0.991, df = 2, N = 116	Little deviation	39 (41.9%) 39.3 (−0.1)	10 (43.5%) 9.7 (0.1)
	Average deviation	25 (26.9%) 24.9 (0.1)	6 (26.0%) 6.1 (−0.1)
	Large deviation	29 (31.1%) 28.9 (0.1)	7 (30.4%) 7.1 (−0.1)
**Eager to learn** χ2 = 0.058, P = 0.971, df = 2, N = 116	Little deviation	33 (35.5%) 32.9 (0.1)	8 (34.8%) 8.1 (−0.1)
	Average deviation	34 (36.6%) 33.7 (0.2)	8 (34.8%) 8.3 (−0.2)
	Large deviation	26 (28.0%) 26.5 (−0.2)	7 (30.4%) 6.5 (0.2)
**Imperturbable (social)** χ2 = 0.078, P = 0.962, df = 2, N = 116	Little deviation	31 (33.3%) 30.5 (0.3)	7 (30.4%) 7.5 (−0.3)
	Average deviation	38 (40.9%) 38.5 (−0.2)	10 (43.5%) 9.5 (0.2)
	Large deviation	24 (25.8%) 24.1 (0.0)	6 (26.1%) 5.9 (0.0)
**Alert** χ2 = 0.707, P = 0.702, df = 2, N = 116	Little deviation	28 (30.1%) 28.1 (0.0)	7 (30.4%) 6.9 (0.0)
	Average deviation	40 (43.0%) 38.5 (0.7)	8 (34.8%) 9.5 (−0.7)
	Large deviation	25 (26.9%) 26.5 (−0.8)	8 (34.8%) 6.5 (0.8)
**Balanced** χ2 = 3.353, P = 0.187, df = 2, N = 116	Little deviation	25 (26.9%) 28.1 (−1.6)	10 (43.5%) 6.9 (1.6)
	Average deviation	37 (39.8%) 33.7 (1.6)	5 (21.7%) 8.3 (−1.6)
	Large deviation	31 (33.3%) 31.3 (−0.1	8 (34.8%) 7.7 (0.1)
**Precise** **χ2 = 15.165, P < 0.001, df = 2, N = 116**	Little deviation	**22 (23.7%)** **29.7 (−3.8)**	**15 (65.2%)** **7.3 (3.8)**
	Average deviation	32 (34.4%) 29.7 (1.2)	5 (21.7%) 7.3 (−1.2)
	Large deviation	**39 (41.9%)** **33.7 (2.6)**	**3 (13.0%)** **8.3 (−2.6)**
**Independent** χ2 = 0.379, P = 0.827, df = 2, N = 116	Little deviation	21 (22.6%) 20.0 (0.5)	4 (17.3%) 5.0 (−0.5)
	Average deviation	24 (25.8%) 24.9 (−0.4)	7 (30.4%) 6.1 (0.4)
	Large deviation	48 (51.6%) 48.1 (0.0)	12 (52.2%) 11.9 (0.0)
**Impulsive** χ2 = 2.620, P = 0.270, df = 2, N = 116	Little deviation	15 (16.1%) 12.8 (1.5)	1 (4.3%) 3.2 (−1.5)
	Average deviation	26 (28.0%) 28.1 (−1.0)	9 (39.1%) 6.9 (1.0)
	Large deviation	52 (55.9%) 52.1 (−0.1)	13 (56.5%) 12.9 (0.1)
**Calm** χ2 = 3.868, P = 0.145, df = 2, N = 116	Little deviation	14 (15.1%) 16.0 (−1.3)	6 (26.0%) 4.0 (1.3)
	Average deviation	35 (37.6%) 31.3 (1.8)	4 (17.4%) 7.7 (−1.8)
	Large deviation	44 (47.3%) 45.7 (−0.8)	13 (56.5%) 11.3 (0.8)
**Flexible** χ2 = 0.354, P = 0.838, df = 2, N = 116	Little deviation	12 (12.9%) 12.8 (−0.6)	4 (17.3%) 3.2 (0.6)
	Average deviation	32 (34.4%) 32.1 (0.0)	8 (34.8%) 7.9 (0.0)
	Large deviation	49 (52.7%) 48.1 (0.4)	11 (47.8%) 11.9 (−0.4)
**Handler-oriented** χ2 = 5.711, P = 0.058, df = 2, N = 116	Little deviation	13 (14.0%) 16.8 (−2.3)	8 (34.8%) 4.2 (2.3)
	Average deviation	52 (55.9%) 50.5 (0.7)	11 (47.8%) 12.5 (−0.7)
	Large deviation	28 (30.1%) 25.7 (1.2)	4 (17.3%) 6.3 (−1.2)
**Careful** χ2 = 9.865, P = 0.007, df = 2, N = 116	Little deviation	12 (12.9%) 11.2 (0.6)	2 (8.7%) 2.8 (−0.6)
	Average deviation	28 (30.1%) 34.5 (−3.1)	15 (65.2%) 8.5 (3.1)
	Large deviation	53 (57.0%) 47.3 (2.7)	6 (26.0%) 11.7 (−2.7)
**Docile** χ2 = 0.064, P = 0.969, df = 2, N = 116	Little deviation	11 (11.8%) 11.2 (−0.2)	3 (13.0%) 2.8 (0.2)
	Average deviation	39 (41.9%) 39.3 (−0.1)	10 (43.5%) 9.7 (0.1)
	Large deviation	43 (46.2%) 42.5 (0.2)	10 (43.5%) 10.5 (−0.2)
**Possessive** **χ2 = 13.528, P = 0.001, df = 2, N = 116**	Little deviation	**11 (11.8%)** **16.8 (−3.5)**	**10 (43.5%)** **4.2 (3.5)**
	Average deviation	26 (28.0%) 22.4 (1.9)	2 (8.7%) 5.6 (−1.9)
	Large deviation	56 (60.2%) 53.7 (1.1)	11 (47.8%) 13.3 (−1.1)
**Excited** χ2 = 1.524, P = 0.467, df = 2, N = 116	Little deviation	11 (11.8%) 12.8 (−1.2)	5 (21.7%) 3.2 (1.2)
	Average deviation	18 (19.4%) 17.6 (0.2)	4 (17.4%) 4.4 (−0.2)
	Large deviation	64 (68.8%) 62.5 (0.7)	14 (60.9%) 15.5 (−0.7)

## Discussion

We aimed to provide better insight into characteristics that are deemed essential for successful professional deployment of scent detection dogs (SDDs) as this may enhance the selection of suitable dogs. As in The Netherlands selection of suitable dogs regards not puppies, but juvenile dogs, we aim to indicate those characteristics that are most relevant to address in future studies on how to measure those characteristics in adolescent or adult dogs, e.g., via behavioral testing. Our starting point, the informal interviews, yielded an extensive list of dog characteristics that were either wanted, unwanted or somewhere in between. Some of these characteristics were deemed more susceptible than others to the effects of training and/or less easily influenced by management during professional deployment. Our second round of structured interviews allowed us to tap into the SDD work experience of 87 handlers and trainers from two professional organizations, who indicated how an ‘ideal dog’ would score on these characteristics. They subsequently indicated how important it was that an actual dog met their ideal image; thus indicating how reluctant they were to make concessions on a certain characteristic through so-called allowed deviation scores. Professional participants gave the lowest allowed deviation score (most reluctant to see deviation from their ideal image) most often to a dog’s characteristics of *scent-oriented* (73.1%), *neurotic* (63.4%), *persistent* (62.4%), *insecure* (55.9%), *resilient* (47.3%), *confident* (45.2%), *independent* (45.2%), and *imperturbable (environment)* (45.2%). This indicates how these characteristics are deemed important as to be present or absent in a certain degree in an SDD dog by professional trainers and handlers. Two of these eight characteristics, *neurotic* and *confident*, overlap with two of the five dimensions of the Monash Canine Personality Questionnaire (MCPQ; extraversion, self-assuredness/motivation, training focus, amicability and neuroticism, N.B. the Dutch term used for ‘confident’ can be translated to either ‘confident’ or ‘self-assured’ [15]. Another two of our eight characteristics, *persistent* and *independent*, are subscales of self-assuredness in the MCPQ. The descriptions that we used for *insecure* overlap with the subscales of neuroticism in the MCPQ. Personality is recognized in dogs among other species and by definition relatively consistently displayed in behavioral responses to stimuli and contexts [16]. Our professional participants were therefore understandably reluctant to see an actual dog deviate from their ideal dog for *neurotic*, *insecure*, *confident*, *persistent*, and *independent*, as these characteristics are regarded part of a dog’s personality and thus not easily influenced by training and/or management, while apparently highly important in SDDs. Professional participants also gave low allowed deviation scores for *resilient* and *scent-orientated*. Leys et al. [17] discussed various issues related to the concept of resilience in humans, which they defined as a dynamic process allowing for positive adaptation in a context of significant adversity. This definition has similarities with the vastly simplified definition we used in the structured interviews: ‘Easily recovers from stress and adversity’. Leys et al. [17] discussed resilience as a personality trait versus a (learned) skill and concluded the determination as one or the other to be impossible at this time. Presumably, our participants would regard resilience to be more a trait than an influenceable skill, given the low allowed deviation scores, indicating their reluctance to have this characteristic deviate from their ideal SDD image. Scent-orientation or ‘intrinsic desire to sniff’ was discussed by five of eight focus groups in a study that, similarly to ours, aimed to define characteristics of successful biosecurity SDDs [18]. Here, it was described as an innate desire to seek and investigate odors and described as an underlying trait for a strong hunt drive or motivation to search. This substantiates our participants’ choice of being reluctant to see this characteristic deviate from their ideal image. Moser et al. [18] also identified ‘low distractibility’ (a subset of ‘focus) as an important working characteristic, consistent with our finding of *imperturbable (environment)* being among the eight characteristics deemed most important.

When comparing the allowed deviation scores for each characteristic between professionals and civilians, we found professionals to be less reluctant than civilians to make concessions on the characteristic *precise* (P < 0.001). For civilians, this characteristic was among the eight for which they were the most reluctant to make concessions. The reason professionals had for giving this characteristic a higher allowed deviation score, was found in the informal interview data. Several professionals described how they improved precision while searching through training, by having the dog search for smaller samples of their trained odor. Dogs with too broad a search tactic are then unable to find the odor and receive their reward; thus having to change their search tactic to be more precise. Thus, our study finds evidence that ‘search precision’ may be regarded as more susceptible to training and/or management; thus possibly not an effective attention point when selecting dogs as SDDs. Professionals were also less reluctant than civilians to make concessions for *possessive* (P = 0.001). This is especially interesting, as professionals desired much higher scores therefor than civilians (52 ± 26.5 versus 14 ± 18.2; P < 0.001). In the informal interviews, professionals indicated the ability to develop possessiveness during training. Civilians, on the other hand, viewed possessiveness as a mainly negative trait, that could be difficult to reduce with training. Another characteristic for which the allowed deviation score differed between civilians and professionals was *unreserved*, with professionals being more reluctant than civilians to make concessions (P < 0.001). The description that we used for *unreserved* overlap with the subscales of neuroticism in the MCPQ, thus the basis of this characteristic lies in personality. Therefore, a dog being more or less *unreserved* is unlikely to be influenced by training and/or management. Due to high performance demands being put on professional SDDs even in situations that are likely to elicit fear or anxiety, professionals will likely more so than civilians see their dogs confronted with working situations that require a certain level of unreservedness. Although not studied here, unreservedness may allow a dog better coping with its surroundings and work task, consequently affecting a dog’s welfare and this factor and a possible positive effect on both work success and animal welfare, may provide an interesting characteristic to address in future studies.

We hypothesized that the characteristics with the lowest allowed deviation scores (the most reluctance to see deviation from the ideal dog) would be the ones with the least variation in the ideal dog scores (how the participant’s ideal dog would score). We found this hypothesis largely confirmed as the least variation within the professionals’ ideal dog scores for the eight characteristics of *neurotic*, *insecure*, *imperturbable (environment)*, *scent-oriented*, *imperturbable (social)*, *confident*, *persistent* and *resilient* overlapped with seven out of the eight characteristics with the lowest allowed deviation scores. For *imperturbable (social)*, professional participants were less reluctant to see deviation from their ideal dog. This means that though they desired a socially imperturbable dog 89 ± 9.1 out of 100), they were willing to work on improving this characteristic through training and/or influence it with management. This seems plausible, since a non-fearful and non-aggressive dog’s reaction to unfamiliar people and animals can to a degree be influenced through operant conditioning and also the working environment can sometimes be modified to limit distractions. Following this reasoning, one would expect *imperturbable (environment)* to also receive slightly higher allowed deviation scores. Possibly, the social distractions are dealt with by management easier than non-social environmental distractions, for instance by cording off search areas. Thus *imperturbable (social)* more so than *independent* is possibly adaptable to some degree via training and/or management. The characteristic of *independent* was scored with more variation, but participants were very reluctant to make concessions. We suspected the variation in ideal score to be due at least partly to different preferences between the disciplines in which the participants work, but we found the discipline to have no effect on the scores. Unfortunately, we found no studies that could help us underpin or explain our findings regarding this variation. A possible alternative explanation is a handler’s or trainer’s personality and/ or working relationship or experience with their present or previous SDDs, but the present data do not allow us to assess these or alternative explanations. We did find that participant gender and year of birth had no effect on the scores. Future studies could assess how variation on these important characteristics could be affected by for instance personal preferences, with for instance some participants preferring to work more closely together with a less independent dog.

We furthermore hypothesized civilian handlers to less frequently score characteristics at the extremes on the 0–100 scale, i.e., be ‘less demanding’, thus resulting in less low means for generally unwanted characteristics and less high means for generally wanted characteristics. This hypothesis is rejected based upon our current results, since only 4 out of 24 characteristics – *unreserved*, *impulsive*, *excited* and *possessive* – differed significantly, of which three confirm the opposite of our hypothesis. Again, we found no studies that could help us underpin or explain our findings. The lack of differences in scores between professionals and civilians could presumably be due to differences in the intensity of the work that these subsamples require from their dogs and a therefrom-resulting difference in their frame of reference. Civilian handlers might think the highest level of a desired characteristic is required in order to perform well at their tasks, while professional handlers are aware that substantially more challenging tasks exist. Alternative explanations could be in a different general management and living situation between person and dog, widening the scope of reference when scoring a dog. Interestingly, the three characteristics that confirm the opposite of our hypothesis and were thus scored more to the extremes by civilians than by professionals, were among the eight characteristics with the largest variation. Also here, discipline, gender and year of birth of the participant did not affect the scores, so the variation in the scores was either due to a factor we did not record, or the result of differences in personal preference within the subsamples of civilians and professionals. The three characteristics were *impulsive*, *excited* and *possessive*, and they were all scored lower by civilians, meaning that they desired a less impulsive, less excited and less possessive dog than professionals did. Impulsivity is generally seen as a trait, which can be measured in dogs using the Dog Impulsivity Assessment Scale (DIAS) [19], though Barela et al. [20] concluded from a systematic review and meta-analysis that there is no good evidence of impulsivity as a behavioral trait that transfers across contexts. They suggest focusing on the context-specific nature of impulsivity in dogs. Civilians desiring a less impulsive dog might be explained by the strong link that was found between impulsivity and owner-reported behavioral problems in pet dogs [19]. The civilians live with their dogs in their homes and the dogs are pets above all, while professional SDDs are kept mostly in kennels when not working. Furthermore, if impulsivity is indeed context-specific, it might benefit professional SDDs in certain tasks. The difference in preferred scores for excited could be explained in a similar way. Arousal (N.B. the Dutch term used for ‘excited’ can be translated to either ‘excited’ or ‘aroused’) was one of the working dog characteristics found [18], whereas for an in-home pet dog, high excitedness/arousal is generally unwanted. For instance, excessive activity was the most commonly mentioned behavioral problem in an Iranian study (38.7% of 345 dogs) [21] and 14.8% of 43,517 dogs showed hyperactive/restless behavior in the US [22]. Lastly, higher possessiveness, which professionals on average preferred, is understandable when this leads to higher reward motivation. Moser et al. [18] found that all eight of their focus groups raised a high reward motivation (strong desire for a given reward) as a desired characteristic. Some of their participants aimed for this desire to be ‘obsessive’. Strong reluctance to give up possessions might turn into problematic resource-guarding for pet dogs. Resource-guarding was defined by Jacobs et al. [23] as ‘the use of avoidance, threatening, or aggressive behaviors by a dog to retain control of food or non-food items in the presence of a person or other animal’, which was understandably mentioned by dog owners as a behavioral problem.

Our study tapped into the knowledge and experience of a relatively large group of dog handlers and trainers. It pinpoints a set of characteristics in SDDs that are likely deemed less susceptible to training and/or management. It also indicates key characteristics that may be different between professionals and civilians working with SDDs. These characteristics can serve as focus point in future studies. Yet, our study has several limitations and must be regarded within its remit and aims. The main limitation is likely in some remaining variation in interpretation of the characteristics and the descriptions by our participants, despite our best efforts to provide precise descriptions and participant instructions. Furthermore, interview techniques, which where necessary to tap into the knowledge and experience, as a methodology may come with certain bias forms. One of the most common sources of bias affecting the validity of interview findings is social desirability [24]. Social desirability is the tendency of participants to answer questions in a way that is viewed favorably by others. This may lead to underrepresentation of answers perceived by the interviewee as incorrect in eyes of the interviewer. Our study would likely be less affected by this form of bias as we had no right or wrong answers and focused on an ‘ideal image’ of the SDD. Other forms of bias however may have affected our participants, which could for instance on the day of an interview have had a certain positive or negative experience with an SDD leading to a momentarily different weighing of SDD characteristics. Also, our study did not involve observations of handler, trainer and dog behavior, which would be a logical future step in studying ideal scent detection dog characteristics for successful professional deployment.

To provide directions for such future studies, a study basis of those SDD characteristics that professional handlers and trainers point to as present in an ideal SDD at a certain level and with a certain importance, is of value. We suggest as a study basis the SDD characteristics of *neurotic*, *insecure*, *imperturbable (environment)*, *scent-oriented*, *independent*, *confident*, *persistent,* and *resilient*, as these were the eight characteristics that received the lowest allowed deviation scores, and were generally scored with very low variation in ideal scores between professionals. Studying these characteristics further can present insights into how the actual SDD pool today differs from the ideal dog characteristics and offers opportunities to enhance SDD selection and training for professional deployment and provide a basis for the development of behavioral tests or specific observations for professional SDD selection in the future.

## Supporting information

S1 File Table A - Multivariate Analysis of Variance (MANOVA) with the participants’ gender, year of birth and number of dogs handled as fixed factors and ideal scores for all 24 characteristics as dependent variables. Regarding p-values <0.0021 as significant, we found no effects. Table B - Multivariate Analysis of Variance (MANOVA) with the discipline as fixed factor and ideal scores for all 24 characteristics as dependent variables. To test for effects of the discipline in which handlers were active, we included disciplines for which we had data from at least ten participants. These were explosives (N = 17), narcotics (N = 14), human scent (N = 19) and currency (N = 13) in the police organization and narcotics (N = 11) in the second professional organization. Regarding p-values <0.0021 as significant, we found no effects. Table C - Multivariate Analysis of Variance (MANOVA) with the participants’ gender, year of birth and number of dogs handled as fixed factors fixed and 1–3 allowed deviation scores for all 24 characteristics as dependent variables. Regarding p-values <0.0021 as significant, we found no effects. Table D - Multivariate Analysis of Variance (MANOVA) with the discipline as fixed factor and 1–3 allowed deviation scores for all 24 characteristics as dependent variables. To test for effects of the discipline in which handlers were active, we included disciplines for which we had data from at least ten participants. These were explosives (N = 17), narcotics (N = 14), human scent (N = 19) and currency (N = 13) in the police organization and narcotics (N = 11) in the second professional organization. Regarding p-values <0.0021 as significant, we found no effects.
(DOCX)
