# Supplementary material for: Exploring ideal scent detection dog characteristics for successful professional deployment as derived from face-to-face structured interviews with professional scent detection dog handlers
Source: PLoS One. 2026 Feb 4;21(2):e0339379. doi: 10.1371/journal.pone.0339379 (PMC12871973; doi:10.1371/journal.pone.0339379)
Supplement: S1 File — Table A - Multivariate Analysis of Variance (MANOVA) with the participants’ gender, year of birth and number of dogs handled as fixed factors and ideal scores for all 24 characteristics as dependent variables. Regarding p-values <0.0021 as significant, we found no effects. Table B - Multivariate Analysis of Variance (MANOVA) with the discipline as fixed factor and ideal scores for all 24 characteristics as dependent variables. To test for effects of the discipline in which handlers were active, we included disciplines for which we had data from at least ten participants. These were explosives (N = 17), narcotics (N = 14), human scent (N = 19) and currency (N = 13) in the police organization and narcotics (N = 11) in the second professional organization. Regarding p-values <0.0021 as significant, we found no effects. Table C - Multivariate Analysis of Variance (MANOVA) with the participants’ gender, year of birth and number of dogs handled as fixed factors fixed and 1–3 allowed deviation scores for all 24 characteristics as dependent variables. Regarding p-values <0.0021 as significant, we found no effects. Table D - Multivariate Analysis of Variance (MANOVA) with the discipline as fixed factor and 1–3 allowed deviation scores for all 24 characteristics as dependent variables. To test for effects of the discipline in which handlers were active, we included disciplines for which we had data from at least ten participants. These were explosives (N = 17), narcotics (N = 14), human scent (N = 19) and currency (N = 13) in the police organization and narcotics (N = 11) in the second professional organization. Regarding p-values <0.0021 as significant, we found no effects. (DOCX) [file pone.0339379.s001.docx]

**S1 Supporting information**

**Table A**

Multivariate Analysis of Variance (MANOVA) with the participants’ gender, year of birth and number of dogs handled as fixed factors and ideal scores for all 24 characteristics as dependent variables. Regarding p-values <0.0021 as significant, we found no effects.

| **Source** | **Dependent Variable** | **df** | **F-value** | **P-value** |
| --- | --- | --- | --- | --- |
| Corrected Model  Fixed Factors  *Participant* *Year of Birth*  *Participant* *Gender*  *Number of dogs handled* | Dependent | 78 | 1.162 | 0.447 |
|  | Alert | 78 | 1.694 | 0.217 |
|  | Possessive | 78 | 1.226 | 0.409 |
|  | Balanced | 78 | 0.739 | 0.769 |
|  | Flexible | 78 | 1.611 | 0.242 |
|  | Handler-oriented | 78 | 3.045 | 0.047 |
|  | Scent-oriented | 78 | 2.360 | 0.096 |
|  | Sensitive | 78 | 3.752 | 0.025 |
|  | Impulsive | 78 | 2.569 | 0.077 |
|  | Calm | 78 | 1.703 | 0.214 |
|  | Eager to Learn | 78 | 2.900 | 0.055 |
|  | Precise | 78 | 1.811 | 0.187 |
|  | Neurotic | 78 | 0.942 | 0.602 |
|  | Unreserved | 78 | 0.196 | 1.000 |
|  | Imperturbable (environment) | 78 | 1.263 | 0.388 |
|  | Imperturbable (social) | 78 | 1.891 | 0.168 |
|  | Insecure | 78 | 0.931 | 0.610 |
|  | Excited | 78 | 2.009 | 0.146 |
|  | Resilient | 78 | 0.772 | 0.742 |
|  | Docile | 78 | 0.712 | 0.792 |
|  | Persistent | 78 | 2.919 | 0.054 |
|  | Careful | 78 | 0.885 | 0.647 |
|  | Independent | 78 | 0.585 | 0.891 |
|  | Confident | 78 | 0.835 | 0.688 |

**Table B**

Multivariate Analysis of Variance (MANOVA) with the discipline as fixed factor and ideal scores for all 24 characteristics as dependent variables. To test for effects of the discipline in which handlers were active, we included disciplines for which we had data from at least ten participants. These were explosives (N=17), narcotics (N=14), human scent (N=19) and currency (N=13) in the police organization and narcotics (N=11) in the second professional organization. Regarding p-values <0.0021 as significant, we found no effects.

| **Source** | **Dependent Variable** | **df** | **F-value** | **P-value** |
| --- | --- | --- | --- | --- |
| Corrected Model  Fixed Factor *Discipline* | Dependent | 4 | 0.223 | 0.925 |
|  | Alert | 4 | 0.851 | 0.498 |
|  | Possessive | 4 | 0.903 | 0.467 |
|  | Balanced | 4 | 0.271 | 0.895 |
|  | Flexible | 4 | 0.255 | 0.906 |
|  | Handler-oriented | 4 | 1.305 | 0.277 |
|  | Scent-oriented | 4 | 0.730 | 0.574 |
|  | Sensitive | 4 | 0.462 | 0.763 |
|  | Impulsive | 4 | 1.572 | 0.191 |
|  | Calm | 4 | 0.869 | 0.487 |
|  | Eager to Learn | 4 | 0.313 | 0.868 |
|  | Precise | 4 | 0.908 | 0.464 |
|  | Neurotic | 4 | 1.432 | 0.233 |
|  | Unreserved | 4 | 0.118 | 0.976 |
|  | Imperturbable (environment) | 4 | 1.129 | 0.350 |
|  | Imperturbable (social) | 4 | 0.264 | 0.900 |
|  | Insecure | 4 | 0.728 | 0.576 |
|  | Excited | 4 | 1.855 | 0.128 |
|  | Resilient | 4 | 0.809 | 0.524 |
|  | Docile | 4 | 1.205 | 0.317 |
|  | Persistent | 4 | 0.763 | 0.553 |
|  | Careful | 4 | 1.630 | 0.176 |
|  | Independent | 4 | 0.505 | 0.732 |
|  | Confident | 4 | 0.232 | 0.920 |

**Table C**

Multivariate Analysis of Variance (MANOVA) with the participants’ gender, year of birth and number of dogs handled as fixed factors fixed and 1-3 allowed deviation scores for all 24 characteristics as dependent variables. Regarding p-values <0.0021 as significant, we found no effects.

| **Source** | **Dependent Variable** | **df** | **F-value** | **P-value** |
| --- | --- | --- | --- | --- |
| Corrected Model  Fixed Factors  *Participant* *Year of Birth*  *Participant* *Gender*  *Number of dogs handled* | Dependent | 78 | 1.369 | 0.336 |
|  | Alert | 78 | 0.752 | 0.758 |
|  | Possessive | 78 | 0.495 | 0.945 |
|  | Balanced | 78 | 1.099 | 0.487 |
|  | Flexible | 78 | 0.866 | 0.663 |
|  | Handler-oriented | 78 | 1.309 | 0.365 |
|  | Scent-oriented | 78 | 0.671 | 0.825 |
|  | Sensitive | 78 | 0.877 | 0.654 |
|  | Impulsive | 78 | 1.369 | 0.336 |
|  | Calm | 78 | 0.557 | 0.910 |
|  | Eager to Learn | 78 | 2.670 | 0.069 |
|  | Precise | 78 | 0.900 | 0.635 |
|  | Neurotic | 78 | 1.327 | 0.356 |
|  | Unreserved | 78 | 1.858 | 0.176 |
|  | Imperturbable (environment) | 78 | 0.570 | 0.901 |
|  | Imperturbable (social) | 78 | 1.637 | 0.234 |
|  | Insecure | 78 | 1.045 | 0.524 |
|  | Excited | 78 | 1.377 | 0.332 |
|  | Resilient | 78 | 1.394 | 0.324 |
|  | Docile | 78 | 3.995 | 0.021 |
|  | Persistent | 78 | 1.324 | 0.357 |
|  | Careful | 78 | 0.561 | 0.907 |
|  | Independent | 78 | 0.859 | 0.668 |
|  | Confident | 78 | 0.575 | 0.897 |

**Table D**

Multivariate Analysis of Variance (MANOVA) with the discipline as fixed factor and 1-3 allowed deviation scores for all 24 characteristics as dependent variables. To test for effects of the discipline in which handlers were active, we included disciplines for which we had data from at least ten participants. These were explosives (N=17), narcotics (N=14), human scent (N=19) and currency (N=13) in the police organization and narcotics (N=11) in the second professional organization. Regarding p-values <0.0021 as significant, we found no effects.

| **Source** | **Dependent Variable** | **df** | **F-value** | **P-value** |
| --- | --- | --- | --- | --- |
| Corrected Model  Fixed Factor *Discipline* | Dependent | 4 | 0.052 | 0.995 |
|  | Alert | 4 | 0.942 | 0.445 |
|  | Possessive | 4 | 0.771 | 0.548 |
|  | Balanced | 4 | 0.947 | 0.442 |
|  | Flexible | 4 | 0.275 | 0.893 |
|  | Handler-oriented | 4 | 0.372 | 0.828 |
|  | Scent-oriented | 4 | 0.973 | 0.428 |
|  | Sensitive | 4 | 0.566 | 0.688 |
|  | Impulsive | 4 | 1.633 | 0.176 |
|  | Calm | 4 | 0.812 | 0.521 |
|  | Eager to Learn | 4 | 0.195 | 0.940 |
|  | Precise | 4 | 2.147 | 0.084 |
|  | Neurotic | 4 | 0.922 | 0.456 |
|  | Unreserved | 4 | 0.370 | 0.829 |
|  | Imperturbable (environment) | 4 | 0.232 | 0.920 |
|  | Imperturbable (social) | 4 | 1.618 | 0.179 |
|  | Insecure | 4 | 0.645 | 0.633 |
|  | Excited | 4 | 0.552 | 0.698 |
|  | Resilient | 4 | 1.017 | 0.405 |
|  | Docile | 4 | 0.237 | 0.917 |
|  | Persistent | 4 | 0.372 | 0.828 |
|  | Careful | 4 | 2.153 | 0.083 |
|  | Independent | 4 | 0.258 | 0.904 |
|  | Confident | 4 | 1.968 | 0.109 |
